# Oral Myiasis Caused by *Cochliomyia hominivorax* in a Disabled Person

**DOI:** 10.1155/2015/904658

**Published:** 2015-07-21

**Authors:** José Pereira Novo-Neto, Fabiano de Sant'Ana dos Santos, Ana Emília Farias Pontes, Fernando Salimon Ribeiro, Fábio Luiz Ferreira Scannavino, Alex Tadeu Martins

**Affiliations:** ^1^Department of Traumatology and Bucco-Maxillofacial Surgery, Santa Casa de Misericórdia de Barretos, 14780-320 Barretos, SP, Brazil; ^2^School of Dentistry, Educational Foundation of Barretos, UNIFEB, 14783-226 Barretos, SP, Brazil

## Abstract

Myiasis is a parasitic disease caused by developing maggots of fly species, which can infect humans. Patients with special needs, especially those with severe neuropsychomotor limitations, may have oral manifestations of this disease. Here, we present a clinical case in which a disabled person was affected by oral myiasis caused by *Cochliomyia hominivorax*. Maggots were found in two ulcerated lesions, a 2 cm diameter lesion in the maxilla and a 6 cm diameter lesion in the mandible. Forty-five maggots were removed during inspection, whereas 75 maggots were surgically removed under general anesthesia with nasotracheal intubation. Dipyrone, ivermectin, and clindamycin were prescribed, and the patient remained hospitalized for 3 days. Seven days after surgical intervention, no maggots were observed. Our study emphasizes that dentists must recognize the symptoms and behaviors of parasitic diseases that affect the oral cavity.

## 1. Introduction

Oral myiasis is a rare condition that is associated with poor oral hygiene, mouth breathing, neurologic deficiency, cerebral palsy, periodontal disease, incompetent lip, anterior open bite, mouth breathing, and debilitated geriatric and alcoholic patients. Individuals residing in tropical, subtropical, and underdeveloped countries may have a higher frequency of fly maggot infestation. Nevertheless, the incidence of this disease in humans is very low [1, 2].

Oral myiasis may be caused by a variety of fly species, including* Chrysomya bezziana*,* Cochliomyia hominivorax*,* Musca domestica*,* Oestrus ovis*, and* Wohlfahrtia magnifica*. Their eggs or maggots are deposited in natural openings of the human body or in places where there is solution of continuity after copulation. When these eggs hatch, they spread parasites that feed on living and necrotic tissues and food scraps. This situation is severely life-threatening to the patient, in addition to causing pain and tissue destruction [1, 3].

Myiasis is usually treated by ivermectin administration that eliminates parasites after their palsy and death, followed by its mechanical removal with forceps or tweezers. Additionally, antibiotics can also be used to avoid the possibility of secondary infections [4, 5]. Patients with neurological, motor, and psychiatric impairment should receive special care from family and/or caregivers, including oral hygiene and the use of oral masks to prevent myiasis by avoiding the exposure of open wounds [2, 6–10].

In a review of current literature, 18 cases of oral myiasis were compiled [1]. Patients treated in Brazil, Iran, India, Hong Kong, Israel, and Oman were included, with age ranging from 9 to 89 years, including 10 males and 8 females. Most common lesion location was the maxilla (13 cases), although mandible, lips, and “right side of face” have also been cited. Cases of* Cochliomyia hominivorax* were only reported in Brazil, but, interestingly, concomitant lesions in maxilla and mandible were not found.

In another recent report, Ribeiro et al. [3] presented a case report of a 97-year-old male, that had intestinal, dermatological, and heart disease that presented a lesion in posterior maxilla and sinus cavity infected by* Cochliomyia hominivorax.* The removal of 110 maggots and 15 teeth was performed, but, unfortunately, the patient died two days after because of severe infection, which emphasized the relevance of an early diagnosis.

Here, we present a clinical case of a disabled person affected by oral myiasis caused by* Cochliomyia hominivorax* who underwent surgery for parasite removal. Two sites were affected, one in mandible and the other in maxilla.

## 2. Case Presentation

**Figure 1:**
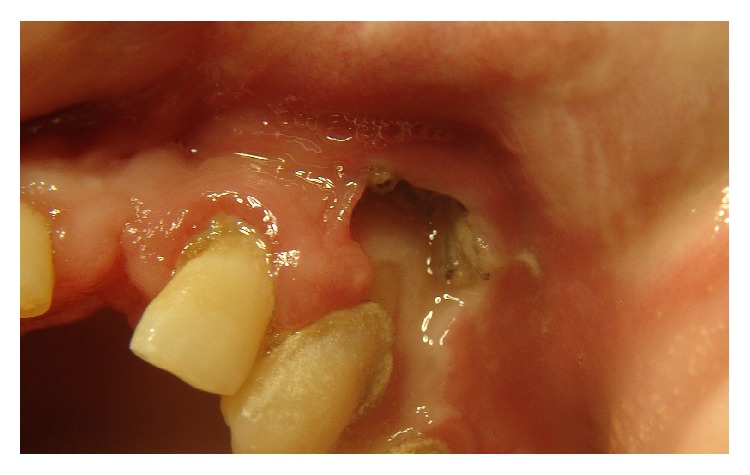
Injury infested with fly maggots in maxilla.

**Figure 2:**
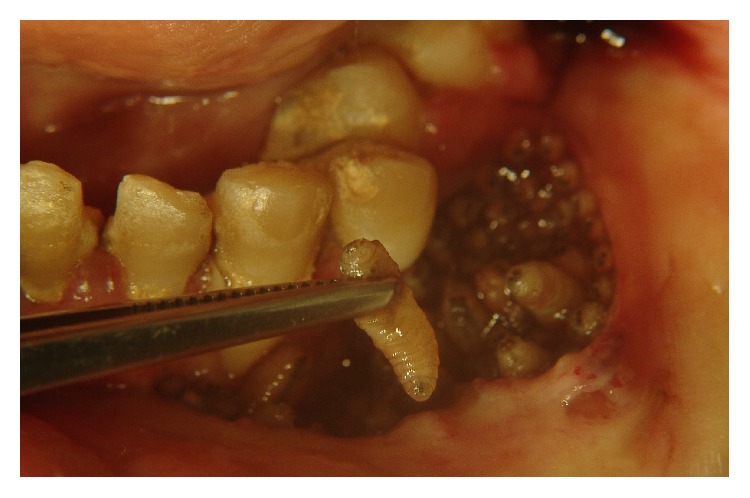
Fly maggots in the mandible.

**Figure 3:**
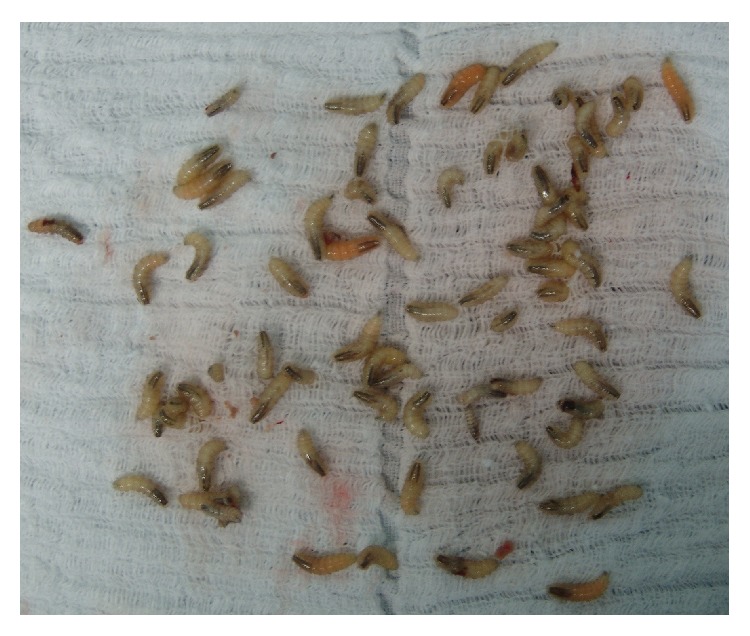
Fly maggots removed from the maxilla and mandible.

A 36-year-old male patient with leukoderma, who resided in a rural area, was brought to the maxillofacial surgery and traumatology clinic by his parent. The parents reported the presence of maggots in the mouth for more than 3 weeks and difficulty in feeding for 2 days. During the examination, we identified congenital blindness, severe psychomotor impairment, and total dependence on his caregiver. The patient used controlled drugs, including diazepam, risperidone, and carbamazepine. Intraoral examination revealed precarious hygiene, halitosis, and generalized severe chronic periodontal disease. In the maxilla, a 2 cm wide, circular, ulcerated lesion with maggots was observed in the left canine region (Figure 1). Furthermore, the jaw on the same side contained a 6 cm, oval, ulcerated lesion full of maggots (Figure 2). During this inspection, 45 maggots were removed with straight forceps, because they were free in the oral cavity. The patient was hospitalized and prescribed 500 mg sodium dipyrone, 600 mg clindamycin, 6 mg ivermectin, and intravenous serum therapy. Surgery to remove the maggots was performed under general anesthesia with nasotracheal intubation, by means of nasal endoscopy. After extra- and intraoral antisepsis, the maggots that afflicted the mandibular mucosa were removed using straight forceps. As there were living maggots, it was necessary to perform surgery to expose, inspect, and remove the maggots that were caged in intramuscular tissue. The chin nerve was broken. The same procedure was performed in the maxilla. The maggots were placed in a flask with 10% formaldehyde. Soft tissues were debrided, and syntheses were carried out to reduce injuries. Seventy-five maggots were removed, of which 67 were confined in the mandibular mucosa. On average, the maggots measured 1.5 cm in length (Figure 3). After the surgery, the patient remained hospitalized for 3 days receiving antibiotics and analgesic medicines. No maggots were present; the patient was fed a pureed diet. Because the patient improved, he was discharged from the hospital, prescribed 5 days of antibiotics, and scheduled for a follow-up analysis of wound healing. His father was advised about the necessity of daily general and special care oral hygiene. Use of a mask was recommended, due to his psychomotor limitations. The maggots were sent to a laboratory for pathological analyses and were identified as* Cochliomyia hominivorax*, based on the morphological characteristics, by its smooth architecture, with no obvious body processes, darkly pigmented dorsal tracheal trunks, and posterior spiracles clearly exposed on last segment.

After 7 days, the patient returned, and an oral tissue inspection revealed that the patient was normal. The patient was referred to the School of Dentistry of the Educational Foundation of Barretos, São Paulo, Brazil, for periodontal treatment for patients with special needs.

## 3. Discussion

The anatomical regions most commonly affected by myiasis are the nose, eyes, lungs, ear, anus, vagina, and mouth [7]. Therefore, this paper is distinguished by the low incidence of this parasitic disease in humans, in particular the oral manifestation of this disease, which requires the knowledge and skill of a dentist [1].

As described in this report, the severe psychomotor limitations presented by the patient, associated with a lack of oral hygiene by the caregiver, certainly contributed to the occurrence of this disease. Halitosis and periodontal disease may have increased the risk of oral myiasis, as reported by other authors [6, 8]. It is reasonable to say that halitosis can attract flies, which deposit eggs or maggots in the gingival tissue affected by chronic periodontal disease. The latter disease can cause intense inflammation, leading to the loss of connective tissues that surround and support the teeth, while the fetid odor of the mouth can attract flies that lay their eggs or maggots in tissues.

However, the patient's father works all day in a commercial area. As a result, oral hygiene after meals was not regularly carried out. After the episode, the father was advised to take care of the patient's daily oral health, thereby preventing periodontal disease, halitosis, and reinfection with fly maggots.

Myiasis can be clinically classified as primary or secondary [9]. In this case, primary oral myiasis was observed, as the maggots were in living tissues and ulcerated due to psychomotor limitations and severe chronic periodontal disease affecting the patient.* Cochliomyia hominivorax* maggots caused extensive tissue destruction [3, 4]. It was evident that the maggots fed quickly on necrotic tissue, causing intense pain and destruction of the mucosa, contributing to disease spread [1–3, 10, 11].

The diagnosis of myiasis is based on clinical evidence, through the visualization of maggots in the tissue. Laboratory analyses are often exempted [4]. In this study, surgical removal of the maggots under general anesthesia was performed because of the patient's special conditions, the extent of the lesions, and the large volume of maggots that needed to be removed. The living maggots were removed and, in both surgical beds (mandible and maxilla), an incision was made to relax the tissue and find the remaining maggots killed by ivermectin before surgery [2, 5, 12]. Importantly, the tissues were extensively damaged, due to the probable mechanical action of the maggots and the toxins they release, as described in the literature [4]. Thus, we removed the necrotic tissues to facilitate repair of the maggot-induced damage, as recommended by other researchers [4, 8].

This report emphasizes the importance of surgeon dentist recognition of myiasis' symptoms, despite its rarity, due to the irreversible health risk.
